# Semi-Supervised Adversarial Auto-Encoder to Expedite Human Activity Recognition

**DOI:** 10.3390/s23020683

**Published:** 2023-01-06

**Authors:** Keshav Thapa, Yousung Seo, Sung-Hyun Yang, Kyong Kim

**Affiliations:** 1 Department of Rehabilitation Medical Engineering, Daegu Haany University, Gyeongsan-si 38610, Republic of Korea; 2 Department of Geriatric Rehabilitation, Daegu Haany University, Gyeongsan-si 38610, Republic of Korea; 3 Department of Electronic Engineering, Kwangwoon University, Seoul 01897, Republic of Korea

**Keywords:** semi-supervised, auto-encoder, human activity recognition, adversarial learning

## Abstract

The study of human activity recognition concentrates on classifying human activities and the inference of human behavior using modern sensing technology. However, the issue of domain adaptation for inertial sensing-based human activity recognition (HAR) is still burdensome. The existing requirement of labeled training data for adapting such classifiers to every new person, device, or on-body location is a significant barrier to the widespread adoption of HAR-based applications, making this a challenge of high practical importance. We propose the semi-supervised HAR method to improve reconstruction and generation. It executes proper adaptation with unlabeled data without changes to a pre-trained HAR classifier. Our approach decouples VAE with adversarial learning to ensure robust classifier operation, without newly labeled training data, under changes to the individual activity and the on-body sensor position. Our proposed framework shows the empirical results using the publicly available benchmark dataset compared to state-of-art baselines, achieving competitive improvement for handling new and unlabeled activity. The result demonstrates SAA has achieved a 5% improvement in classification score compared to the existing HAR platform.

## 1. Introduction

Embedded wearable inertial sensors permit unobtrusive and regular monitoring, making human activity recognition an ideal platform for health assessment [1], predicting depression, cognitive and mental states [2], and monitoring sleep and fitness [3]. Applications for HAR systems in the actual world include smart homes [4], defenses [5], astronauts [6], senior care [7], and defense applications. However, as they must now take into account all unexpected changes in the real-time scenario, the current approaches face significant difficulties in accurately recognizing activities. Modern gesture recognition systems have great accuracy and are based on shallow or deep neural network (DNN) models [8]; however, they still have a significant problem. The pre-trained ML models are strong enough to handle instance-specific differences in the sensor data due to user diversity and their changing activity schedules. It is impractical for actual societal-scale deployment to use the standard method for addressing such heterogeneity, which involves using instance-specific labeled data to develop individual classifiers. Instead, substantial research has concentrated on automated domain adaptation methods, requiring no labeled training data. The majority of HAR machine learning methods, including k-nearest neighbor [9], decision trees [10], and support vector machines [11] in the literature, rely on heuristic feature extraction to build their models. For 3D sensors, this comprises correlation (Pearson correlation) between axes, mean and standard deviation for each sensor signal, and time-domain calculations. The use of transfer learning to alter a training domain model with only modest amounts of labeled data from the test domain has been proposed in recent years [12]; the mapping of domain-dependent sensor values to a domain-independent, common low-dimensional latent space [13]; and the use of adversarial learning. At the moment, efforts at HAR are mainly directed toward learning actively—acquiring user comments about new activities, and detecting changes—finding new activities [14,15,16]. The model must be rebuilt and retrained when adding a new activity class. Few studies have looked into the potential for an activity model to emerge automatically with various activities [17]. However, this capability has the advantage of maintaining the knowledge in the time-tested business model while reducing the need for manual feature engineering, manual configuration, and training expenses.

Several supervised [18] and semi-supervised [19] approaches to activity recognition exist. These models offer good accuracy when given enough training data. However, their performance suffers when applied to novel and unexplored distributions. Therefore, it is still difficult for the model to identify a new user’s activities. Most machine learning [14] and deep learning are not conceptually aware of all activities, but with the proper learning and models, they may effectively recognize human behavior. Many artificial intelligence models and cutting-edge techniques [20] are based on deep neural networks.

On the other hand, deep learning needs a lot of data to serve as a label for learning. The main disadvantage of HAR is that each sensor must be installed and controlled separately. Additionally, the domain’s experts should only understand and label unlabeled data, increasing the labeling task. As a result, our strategy ensures that the activity detection needs are primarily met by better performance than prior methods. This research focuses on semi-supervised adversarial learning, which combines adversarial learning, deep learning, and semi-supervised learning (VAE) to ensure that no labels based on previously learned data can be fully expected. Additionally, there is a chance that this method could enhance speed by employing fewer tagging classes. The advantages of our approach are as follows:

1. We proposed a semi-supervised model that can adapt without labeled data or changes in the pre-trained classifier to identify human activity.
2. Demonstration of adversarial autoencoder (AAE) efficacy and robustness, so that the model will be able to comprehend fresh modifications, which are all inescapable in real-world scenarios.
3. The suggested joint model can directly and automatically structure and learn spatiotemporal characteristics from the unprocessed sensor data without requiring manual feature extraction.
4. This technique may be the most effective state-of-the-art and can probably be used across various platforms and domains.

The remaining paper sections are arranged as follows. Section 2 contains related work. The materials and procedures used in the proposed strategy are illustrated in Section 3. Our experimental setup for the activity recognition method is covered in Section 4 of this article. In Section 5, the activity recognition performance analysis is explained. Finally, a conclusive summary is provided in Section 6.

## 2. Related Work

HAR refers to a set of techniques used to automatically identify the task humans are executing by examining the video, readings from wearable sensors, or wireless signals reflected by the human body [21]. Shallow learning and deep learning techniques can categorize the HAR algorithms. SVM [22], k-nearest neighbors (kNN) [23], linear discriminant analysis (LDA) [24], and random forest [25] are examples of popular shallow HAR approaches. By learning to extract features from raw sensor data automatically, deep learning approaches, such as LSTM [26], CNN [27], convLSTM [28], and CNN-LSTM [29] have demonstrated impressive improvements in performance compared to their shallow counterparts. These eliminate the need for human experts to provide hand-engineered features.

The decline of cross-subject performance and change in activity schedule [30] is a significant obstacle when using deep learning for HAR. When testing the trained deep learning models on individuals not included in the training set, the difference in data distribution between the training and testing sets frequently results in considerable performance degradation since different subjects carry out the same tasks in different ways [31]. An ideal training set would consist of data collected from tens or even hundreds of additional participants in order to address this problem. However, gathering and classifying data is a tedious and time-consuming operation.

Domain adaption techniques are rooted in natural language processing and computer vision [32] and have recently drawn increased attention for HAR applications [33]. These strategies can be classified as shallow or deep models depending on the feature extraction technique. Transfer learning strategies aim to align statistics of particular features between the source and destination domains for shallow models. Although DNN-based techniques use intermediate representations that are automatically learned by DNNs rather than manually created features, they nevertheless aim to achieve feature alignment. Several adversarial learning-based methods have recently tried to implement the new activity detecting process [34] by automatically identifying characteristics unaffected by the domain mismatch and (ii) useful for categorizing a particular activity. Model ensemble and feature concatenation have been proposed in earlier works [35]. for multi-sensor fusion [36]. However, these works combine a predetermined set of sensors without considering scenarios in which the fusing of several sensor configurations is required. Missing data imputation is a common issue focusing on completing the input space’s unobserved activity data. Wearable sensor data typically involves multiple body locations over an extended time period and is highly dimensional. Furthermore, the majority of deep-learning based categorization models for this type of data offer little to no interpretability for the expected result. Some progress has been made in the challenge of video-based action recognition [37]. The auto-encoder is one class of neural networks capable of learning a condensed representation of the input signals. Ref. [38] suggested encoding high-dimensional continuous data as low-dimensional data by using auto-encoders with several hidden layers, so the features are retrieved. Ref. [39] features LightGBM as the classifier with stacking denoising auto-encoder for feature extraction. For instance, the learned features can be stacked using a stacked auto-encoder, which can then be used to create a classification model [40]. In their continuous auto-encoder proposal, unsupervised outlier detection can also be carried out using ensemble learning. To save on computing costs, ref. [41] presented an ensemble auto-encoder randomly connected with various architectures and connection densities.

## 3. Materials and Proposed Method

**Figure 1:**
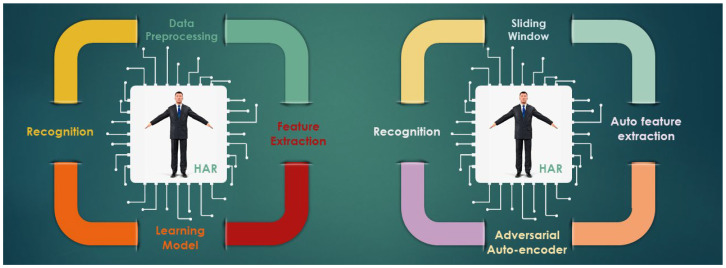
System workflow of our proposed method for HAR.

Systems for recognizing human activity go through data collection, pre-processing, feature extraction, training, and recognition. A similar procedure is also used in our approach, but the motivating aspect is new to HAR as of yet. Figure 1 depicts the process flow for our suggested technique. First, segmentation and filtration are used to pre-process the sensor data. Then, the feature is automatically extracted as we employ the deep learning model. The activity is then trained, classified, and recognized. Finally, we reprocessed and categorized activity that was not annotated. Semi-supervised learning is the term for this method. The auto-encoder model is what we are employing for this method. As a mechanism for adversarial learning, we add some perpetuation to the network to help it create its immune system. So, using the auto-encoder model and semi-supervised adversarial learning, we describe a method to identify human activities.

### 3.1. Semi-Supervised Learning

Learning data and labels under supervision is a technique used in many situations or domains. When solving complex problems, supervised learning employs labeled data to learn and gain knowledge [42]. The supervised learning process has been implemented using a variety of deep learning and machine learning techniques. To train, however, hundreds to millions of learning data points may be given, and categorizing each point is crucial. Due to these problems, supervised learning cannot be applied in the absence of enough learning data. This problem can be solved by semi-supervised learning. It is a method for identifying unlisted data with crucial criteria, such as thresholds, and re-learning models using learning data that is already accessible to improve performance based on the anticipated values of the learned sequences. The semi-supervised approach lessens manual annotation while creating a self-learning model that eventually builds up a solid body of knowledge and improves the recognition model’s efficiency or accuracy. The collection of n labeled data points.

(1) $$d_{N}={\left(x_{i},y_{i}\right)}_{i=1\ldots \ldots n}^{l}$$

$x_{i},\;y_{i}$ consists of data $x_{i}\in ℜ$ from a given sensor space ℜ. We also have access to the extraction of m data whose labels are unknown.

(2) $$d_{M}={\left(x_{i}\right)}_{i=l+1\ldots \ldots l+m}^{l+u}$$

### 3.2. Auto-Encoder

A fundamental AE is a neural network model in which the output replicates the input. The encoder and decoder are the two components that make up an AE. The encoder develops the ability to condense the inputs into a smaller subset of encoded features, or the bottleneck. The decoder learns how to recreate the original input given the encoded features. Consequently, an AE’s output is a rough reconstruction of its input. Formulating the encoder phase is Equation (3), where W is the weight matrix and *b* is the bias vector for the encoder phase. The decoder phase is expressed in Equation (4), for the labeled data. Furthermore, Equations (5) and (6) represent the unlabeled data. *W* is the weight matrix and *b* is the bias vector. In this work, the sigmoid function is used as the nonlinear activation function denoted by the letters *f* in Equations (1) and (2). In the paragraphs that follow, we define x, h and $\bar{x}$ as input layer, hidden layer and output (is approximately the same as the output of x), respectively.

(3) $$h_{d_{N}}=f\left(W_{i}d_{N}\right)+b_{i}$$

(4) $${\bar{x}}_{d_{N}}=f\left(W_{i}h_{d_{N}}\right)+b_{i}$$

(5) $$h_{d_{m}}=f\left(W_{i}\;d_{N}\right)+b_{i}$$

(6) $${\bar{x}}_{d_{m}}=f\left(W_{i}h_{d_{m}}\right)+b_{i}$$

### 3.3. Adversarial Learning

With the addition of minute disturbances or noises to the training data, adversarial learning is a method to regularize neural networks that enhances their ability to predict the future or approaches to deep learning by increasing the loss of a more profound learning model. However, according to [43], even minor changes to the deep learning input could produce very confident wrong decisions. Therefore, the following terms are added by adversarial learning to its cost function during the training phase of a predictive model where x and y are the input and two distinct parameters.

(7) $$\log p\;(Y_{t}^{s}|X_{t}^{s}+r_{t}^{s};\theta )=where\;r_{t}^{s}=arg\min\log p\;(Y_{t}^{s}|X_{t}^{s}+r_{t}^{s};\hat{\theta })$$

According to Equation (7), *r* in the input data is hostile. A set of the recognition model’s constant parameters $\hat{\theta }$ is inherited from the θ. The suggested algorithm recognizes the worst-case perturbations $r_{t}^{s}$ at each training. In opposition to the present trained model, adversarial training generates disturbances or random noise that are easily misclassified in the learning model by changing the input instances, in contrast to other regularization strategies such as dropout.

The Algorithm 1 illustrate the pseudo-code for the overall process of our proposed method. Based on this algorithm we performed the experiment using the python coding.
**Algorithm 1:** Semi-supervised auto-encoder model with adversarial trainingStep 1. Initialize the network Step 2. Reset: inputs = 0, activations = 0 Step 3. Initialize the inputs Step 4. Create encoder and decoder
$h_{d_{N}}=f\left(W_{i}d_{N}\right)+b_{i}$ ${\bar{x}}_{d_{N}}=f\left(W_{i}h_{d_{N}}\right)+b_{i}$
Step 5. Predict and calculate the loss function
Calculate seq2seq lossCalculate class loss using cross-entropy
Step 6. Add random perturbations,
$\log p\;(Y_{t}^{s}|X_{t}^{s}+r_{t}^{s};\theta )=where\;r_{t}^{s}=arg\min\log p\;(Y_{t}^{s}|X_{t}^{s}+r_{t}^{s};\hat{\theta })$
Step 7. Calculate loss function by adding adversarial loss Step 8. Optimize the model based on AdamOptimizer Step 9. Recognize unlabeled data based on Algorithm 1
$h_{d_{m}}=f\left(W_{i}\;d_{N}\right)+b_{i}$ ${\bar{x}}_{d_{m}}=f\left(W_{i}h_{d_{m}}\right)+b_{i}$
Step 10. Add recognized dataset to original training dataset Step 11. Retrain the model


## 4. Experimental Configuration

This section presents the complete results for both training and recognition. The assignment and processing of numerous design hypotheses come first. The proposed model is then trained using labeled and unlabeled data; the outcomes are compared to the outputs of the currently existing models. Finally, the experimental examination of the suggested approach is carried out using the CASAS dataset. This publicly available dataset can be downloaded free from the UCI Machine learning Repository.

To obtain the HAR dataset, a series of tests were run. For this work, 30 individuals with ages ranging from 19 to 48 were chosen. Each participant was given instructions on how to conduct themselves while sporting a Samsung Galaxy S II smartphone on their waist. The six chosen ADLs were *walking, walking upstairs and downstairs, sitting, lying down, and standing*. Each participant went through the process twice: on the first trial, the smartphone was fixed to the left side of the belt; on the second, the user chose where to put it. Additionally, there is a 5 s break between each task where people are instructed to rest. This promotes repeatability (every activity is attempted at least twice) and relaxation.

### 4.1. Parameter Setting

The suggested technique was trained and tested using scikit-learn and the TensorFlow GPU1.13.1 library. The resulting data was pre-processed and sampled in sliding windows that overlapped and had a fixed width of 200 ms and a window length that ranged from 0.25 s to 7 s. Our technique was tested using an i7 CPU with 16 GB of RAM, a GTX Titan GPU running on CUDA 9.0, and the cuDDN 7.0 library. To use as little memory as possible, the CPU and GPU were utilized. A training set, a validation set, and a testing set comprised the three components of the dataset. The remaining 30% of data was used for testing, with the remaining 70% going toward training. The k-fold CV was used to validate the data (cross-validation). To verify, we employed 10-fold cross-validation (K = 10).

(8) $$V=\frac{1}{p}\sum_{p=1}^{10}E\;\left(error\right)$$

In order to reduce overfitting, the dropout rate was adjusted during training to 0.5, removing unneeded neurons from each hidden layer. Training loss can also be decreased by using random initialization and optimizing training parameters. Cross-entropy and L2 normalization were incorporated to prevent overfitting and make the model stable.

(9) $$L=-\frac{1}{k}\sum_{k=1}^{n}y_{t}^{m}\cdot logy_{t}^{s^{\prime }}+\Gamma \cdot \|W\|,$$

where W stands for the weighting parameter and k for the batch size, the label is $y_{t}^{m}$; and the recognized output is $y_{t}^{m}$. By reducing the amount of the weighting parameters, L2 normalization avoids overfitting. Adding minute disturbances or noises to the network with training data increases the loss of a more profound learning model for regularization that improves the recognition ability. Adversarial training is a technique for regularizing neural networks that enhances the neural network’s prediction performance and may even approach deep learning. If the adversarial input is given by $r_{t}^{s}$, then the perturbations are given by, which is written as

(10) $$r_{t}^{s}=arg\min\log p\;(Y_{t}^{s}|X_{t}^{s}+r_{t}^{s};\hat{\theta })$$

In order to achieve the most significant performance, we aimed to select the optimal hyperparameters, such that the learning rate, L2 weight, and difference all decreased. We utilized a learning rate of 0.005 with a batch value of 100 for each epoch to train the model. Learning begins at 0.001. The training is completed when the outputs are stable, which takes about 12,000 epochs. The Adam optimizer is a parameter-free adaptive moment estimator that produces adaptive learning rates. There are two dimensions: 128 for the input and 256 for the output, and 8 hidden layers. Gradient clipping was changed to 5 to lower the gradient crossing threshold. An Adam optimizer was used.

### 4.2. Evaluation Parameter Setting

The model’s performance was assessed using accuracy, *F*1-*score*, and training duration. The confusion matrix, where the row denotes the anticipated class and the column indicates the actual class, can be used to calculate these. The computational recognition accuracy of human activity recognition was assessed using the precision and recall parameters. The percentage of correctly identified instances from perceived activity occurrences is known as precision. A recall is the percentage of cases that were identified adequately out of all the instances. The weighted average of precision and recall between [0, 1] is known as an *F*-score, and a number closer to 1 indicates the more incredible performance

(11) $$Precision\;:\frac{1}{N}\;\left(\overset{N}{\underset{m=1}{\sum }}\frac{TP_{m}}{TP_{m}+FP_{m}}\times 100\right)$$

(12) $$Recall\;:\frac{1}{N}\;\left(\overset{N}{\underset{m=1}{\sum }}\frac{TP_{m}}{TP_{m}+FN_{m}}\times 100\right)$$

(13) $$F-score\;:\frac{2\times Precision\times Recall}{Precision+Recall}$$

(14) $$Accuracy=\frac{1}{N}\;\left(\overset{N}{\underset{m=1}{\sum }}\frac{TP_{m}+TN_{m}}{TP_{m}+TN_{m}+FP_{m}+FN_{m}}\times 100\right)$$

Through the confusion matrix, these terminologies were evaluated for true positive (TP), false positive (FP), and false-negative (FN) results. Each dataset was divided into three groups: a training set, a validation set for parameter optimization, and a test set for final assessment.

## 5. Activity Recognition Results, Analysis and Evaluation

The experimental findings are discussed and examined in this section. The dataset was used to locate all actions. In the context of a smart home, the contemporaneous and interspersed activities that happen the most frequently are referred to as presiding activities. Concurrent activity recognition, interleaved activity recognition, and the recognition average are the three sections that make up the analysis section. The general recognition accuracy is then contrasted with the other state-of-the-art approaches, including the CNN [44], the long short-term memory (LSTM) [45], and synchronized long short-term memory (Syn-LSTM) [46].

**Figure 2:**
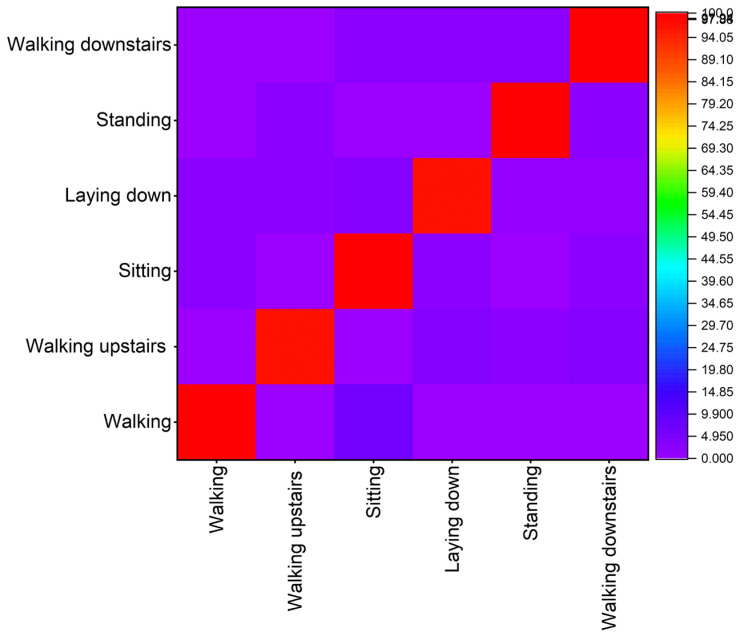
Confusion matrix accuracy.

The recognition confusion matrix of the proposed method is shown in Figure 2. According to the confusion matrix, the average *F*-score for recognition is over 0.98, indicating that the average accuracy is high and desirable.

As previously said, the accuracy is generally decent despite the sparse data. The walking activity to be true is 98% walking upstairs and walking downstairs is 97% and 98%, respectively. The sitting and the lying down possess similar accuracy with 97% and 96%. The sitting and lying down signal changes are very similar until and unless going into depth-hidden layers. Standing activity recognition accuracy is almost 99%. Walking and walking upstairs/downstairs are inherently more accessible to recognize than other activities as their signal changes are more or equal to the walking threshold. This dataset only has limited data and instances, so it is a bit easy to recognize, and accuracy is high enough, i.e., a large number of datasets could obtain the actual and accurate recognition distribution. The main aim of the proposed method is to recognize human activity and find and prove the algorithm.

**Figure 3:**
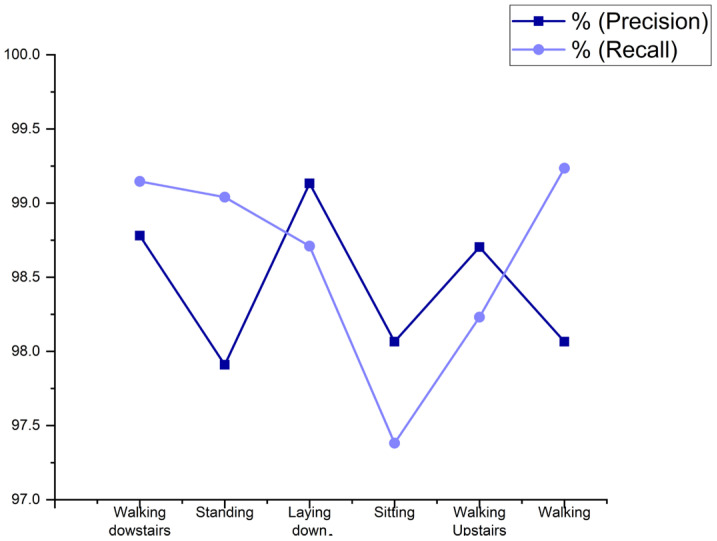
The precision and the recall on the given set of data.

Regarding analysis, the proposed algorithm is more reliable and competent than the existing method. The Figure 3 represents the percentage of cases that were identified correctly as activity instances. The precision of 98.4420% and recall of 98.6231% is received from the proposed model to determine the activity correctly.

We have performed the validation to minimize the error and remove the unwanted activity that leads to increase accuracy. The 10-fold cross-validation is used for the model validation, whose deviation is measured referring epochs and batch sizes. Table 1 displays the mean and standard deviation after 10,000 iterations of changing the batch size hyperparameter. Similar results are shown in Table 2 for the epochs parameter when the batch size is 100, and the mean and standard deviation are computed. The choice of window size is also important for system accuracy; technically, a window size range of 500 ms to 5000 ms will be useful. Table 3 displays the mean and standard deviation of accuracy and error. 

**Figure 4:**
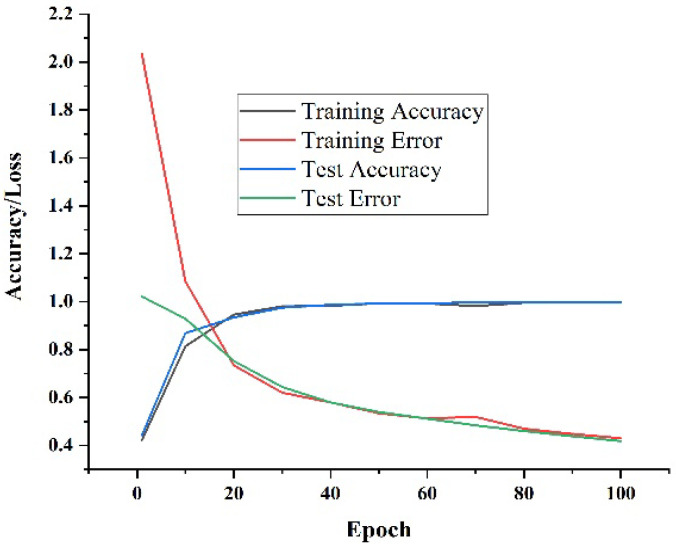
Accuracy and loss curve of training and testing.

**Figure 5:**
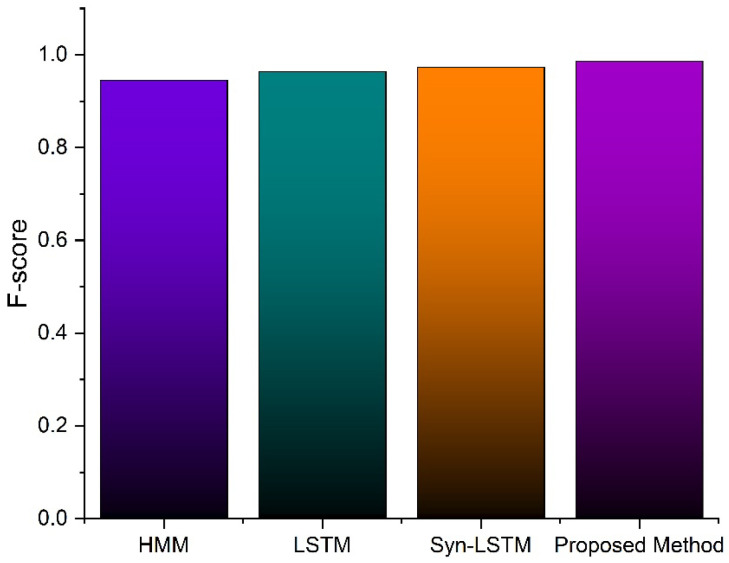
*F*1-*Score* comparison with an existing method.

Figure 4 depicts the accuracy and loss curve. The graphs’ relatively small difference between training and testing accuracy demonstrates the model’s efficacy. The dropout approaches, adversarial training, and semi-supervised learning are advantageous since the difference between training and test loss is also relatively small: Method 98.34, as proposed with an average inaccuracy of 0.1571, had an average accuracy of 98.154%, illustrating the effectiveness of the suggested strategy in comparison to the current framework, including the HMM [33], LSTM [34], and sync-LSTM [35] techniques (algorithms). The *F*1-*score* is higher than 0.98, as shown in Figure 5. Although the sync-LSTM is equally accurate to our approach, it is unable to handle fresh or unannotated data.

## 6. Conclusions

By thoroughly comparing a semi-supervised adversarial auto-encoder with recently introduced activity recognition techniques such as deep learning and its variants, the work presented in this paper demonstrates a workable solution for detecting human activities. However, these strategies do not address the novel and new data in the sequence. On annotated and routine activity detection, many methods have been studied. Few of them, nevertheless, have made an effort to find intricate and unannotated activity. The proposed method recognized unannotated human behavior from the data gathered from the sensors by semi-supervised learning capability. The adversarial learning technique improves learning capacity by introducing slight disturbances or noises to the network. The challenges in recognizing human activity still include accuracy, processing complexity, complex activity, and unannotated activity. Nevertheless, the accuracy is 98%, and the precision and recall are also high, yielding an f1 score of greater than 0.98.

However, due to sensor timing, noise interference, and limited data, the accuracy is not equal or tends to be 100%. The current best-performing model encounters many real-time difficulties while interacting with various datasets. Essential factors affecting model performance include the number of activities carried out, sensor kinds, sensor deployment, population size, and time periods. Since tiny windows might not contain all the information and wide windows might result in overfitting and overload, window size also significantly impacts model performance. Identifying and processing the unannotated data is advantageous for extremely unbalanced datasets.

The suggested method uses reduced pre-processing time and manual feature extraction to automatically extract spatio-temporal information and identify unannotated activity. The proposed method can be improved and upgraded in the future to distinguish more complicated, multiuser, and multivariate actions. Additionally, we may benefit from edge computing, cloud computing, and IoT services to process a lot of data efficiently. Finally, different settings and domains, such as sign language identification, cognitive capacities, etc., can be employed in our approach. As a result, the strategy we recommend is a better, state-of-the-art one for HAR.

## Figures and Tables

**Table 1:** Mean and standard deviation of variable range batch size on 10,000 epochs.

Epochs	1000	5000	8000	10,000
	Mean (µ) ± SD (σ)	Mean (µ) ± SD (σ)	Mean (µ) ± SD (σ)	Mean (µ) ± SD (σ)
	0.9300 ± 0.0125	0.9245 ± 0.0321	0.9386 ± 0.0621	0.9421 ± 0.0221

**Table 2:** Mean and standard deviation of different epochs on 100 batch size.

Batch Size	10	30	60	100
	Mean (µ) ± SD (σ)	Mean (µ) ± SD (σ)	Mean (µ) ± SD (σ)	Mean (µ) ± SD (σ)
	0.9561 ± 0.065	0.9602 ± 0.0423	0.9631 ± 0.04125	0.9531 ± 0.0431

**Table 3:** 10-fold cross-validation result.

	Mean (µ) ± SD (σ) Accuracy	Mean (µ) ± SD (σ) Error
UCI	0.9410 ± 0.0522	0.3215 ± 0.0121

## Data Availability

Not applicable.
